# The effect of passive mobilization associated with blood flow restriction and combined with electrical stimulation on cardiorespiratory safety, neuromuscular adaptations, physical function, and quality of life in comatose patients in an ICU: a randomized controlled clinical trial

**DOI:** 10.1186/s13063-021-05916-z

**Published:** 2021-12-30

**Authors:** Thaís Marina Pires de Campos Biazon, Cleiton Augusto Libardi, Jose Carlos Bonjorno Junior, Flávia Rossi Caruso, Tamara Rodrigues da Silva Destro, Naiara Garcia Molina, Audrey Borghi-Silva, Renata Gonçalves Mendes

**Affiliations:** 1 Cardiopulmonary Physical Therapy Laboratory, Department of Physical Therapy, Federal University of São Carlos, Rod. Washington Luiz, km 235 – SP 310, CEP 13565-905, São Carlos, Brazil; 2 Laboratory of Neuromuscular Adaptations to Resistance Training, Department of Physical Education, Federal University of São Carlos, São Carlos, Brazil; 3 Department of Medicine, Federal University of São Carlos, São Carlos, Brazil; 4 Department of Anesthesiology and Intensive Care Unit at the Irmandade da Santa Casa de Misericórdia de São Carlos, São Carlos, Brazil

**Keywords:** Blood flow restriction, Electrical stimulation, Cardiorespiratory safety, Intensive care unit-acquired atrophy, Intensive care unit-acquired weakness, Neuromuscular adaptations, Physical function, Quality of life, Critical care

## Abstract

**Background:**

Intensive care unit-acquired atrophy and weakness are associated with high mortality, a reduction in physical function, and quality of life. Passive mobilization (PM) and neuromuscular electrical stimulation were applied in comatose patients; however, evidence is inconclusive regarding atrophy and weakness prevention. Blood flow restriction (BFR) associated with PM (BFRp) or with electrical stimulation (BFRpE) was able to reduce atrophy and increase muscle mass in spinal cord-injured patients, respectively. Bulky venous return occurs after releasing BFR, which can cause unknown repercussions on the cardiovascular system. Hence, the aim of this study was to investigate the effect of BFRp and BFRpE on cardiovascular safety and applicability, neuromuscular adaptations, physical function, and quality of life in comatose patients in intensive care units (ICUs).

**Methods:**

Thirty-nine patients will be assessed at baseline (T0–18 h of coma) and randomly assigned to the PM (control group), BFRp, or BFRpE groups. The training protocol will be applied in both legs alternately, twice a day with a 4-h interval until coma awake, death, or ICU discharge. Cardiovascular safety and applicability will be evaluated at the first training session (T1). At T0 and 12 h after the last session (T2), muscle thickness and quality will be assessed. Global muscle strength and physical function will be assessed 12 h after T2 and ICU and hospital discharge for those who wake up from coma. Six and 12 months after hospital discharge, physical function and quality of life will be re-assessed.

**Discussion:**

In view of applicability, the data will be used to inform the design and sample size of a prospective trial to clarify the effect of BFRpE on preventing muscle atrophy and weakness and to exert the greatest beneficial effects on physical function and quality of life compared to BFRp in comatose patients in the ICU.

**Trial registration:**

Universal Trial Number (UTN) Registry UTN U1111-1241-4344. Retrospectively registered on 2 October 2019. Brazilian Clinical Trials Registry (ReBec) RBR-2qpyxf. Retrospectively registered on 21 January 2020, http://ensaiosclinicos.gov.br/rg/RBR-2qpyxf/

**Supplementary Information:**

The online version contains supplementary material available at 10.1186/s13063-021-05916-z.

## Background

Intensive care unit (ICU) therapies that are used to stabilize severe illnesses, such as sedatives, invasive access, and mechanical ventilation are responsible for prolonged lengths of bed restriction and, consequently, for reducing mobility in ICU [1–6]. In turn, mobility restriction is associated with losses in muscle mass and strength [4, 7–13].

ICU-acquired atrophy begins within the first 48 h of severe disease [14–16] and increases during the first two weeks of intensive care [15–17]. Studies suggest that severely ill patients may lose from 25 to 40% of peripheral muscle strength in two or more days of invasive mechanical ventilation [18–20]. During the first week of intensive care, there is evidence of associations between atrophy and weakness with high mortality rates [21–24] and, interestingly, even after discharge from the ICU with reduced physical function [25], higher functional dependence [26–28], and a reduction in quality of life [29–31].

Passive mobilization (PM) has been applied as a therapeutic strategy to reduce low mobility in patients unable to perform active exercises due to sedation [32] or non-induced comatose state [33], but without evidence of muscle atrophy and weakness prevention. Neuromuscular electrical stimulation [7, 8] is another strategy applied in critical care, involuntarily evoking muscle contractions through low-tension electric pulses [34]. Despite this, the efficacy of electrical stimulation still appears to be inconclusive, especially for severely ill patients [35, 36].

Barbalho et al. [37] investigated the use of blood flow restriction (BFR) associated with PM (BFRp) in comatose elderly patients in ICUs. This method consists of controlled mechanical compression to the muscle blood vessels through a pneumatic cuff [38], aiming to reduce arterial supply and muscle venous return and, consequently, stimulating muscle protein synthesis. In an intra-subject design study, both groups (PM and BFRp) presented muscle atrophy, although the application of BFR in one of the limbs efficiently reduced the loss of muscle mass. There were no adverse events, and the authors suggested BFRp as an effective strategy to reduce the magnitude of muscle mass loss.

Abe et al. [39] highlighted the intensity of exercise associated with BFR as an important aspect to optimize neuromuscular adaptations. Thus, Gorgey et al. [40] demonstrated a 17% increase in muscle mass after BFR combined with electrical stimulation in spinal cord-injured patients, compared to isolated electrical stimulation. Given the impossibility of performing active exercises, it seems also necessary to investigate the effects of BFR associated with PM and combined with electrical stimulation (BFRpE) in comatose patients to prevent muscle atrophy and weakness and possibly to optimize physical function and quality of life in ICU survivors.

Regarding BFR safety, after the release of between-sets BFR, rapid local muscular blood inflow and bulky venous return occur [41], which can lead to unknown repercussions in the cardiovascular system [42]. Consequently, these repercussions may reflect in critical changes in vital signs of severely ill patients and therefore in health recovery.

Hence, the present study aimed to investigate the effect of BFRp and BFRpE on cardiovascular safety and applicability, neuromuscular adaptations, physical function, and quality of life in ICU coma patients. We hypothesized that there will be no negative cardiovascular repercussions of BFRp, BFRpE, and BFRp will be able to reduce and BFRpE to prevent muscle atrophy and weakness, in addition to exerting more positive effects on physical function and on quality of life compared to BFRp.

## Methods

### Primary objectives

The primary objectives are to investigate the effect of BFRp and BFRpE on cardiovascular safety in comatose patients in the ICU and to investigate the applicability of such protocols as early motor intervention methods in intensive care.

### Secondary objective

The secondary objective is to investigate the effects of this intervention on neuromuscular adaptations, physical function, and quality of life of ICU coma-surviving patients.

### Study design

**Fig. 1:**
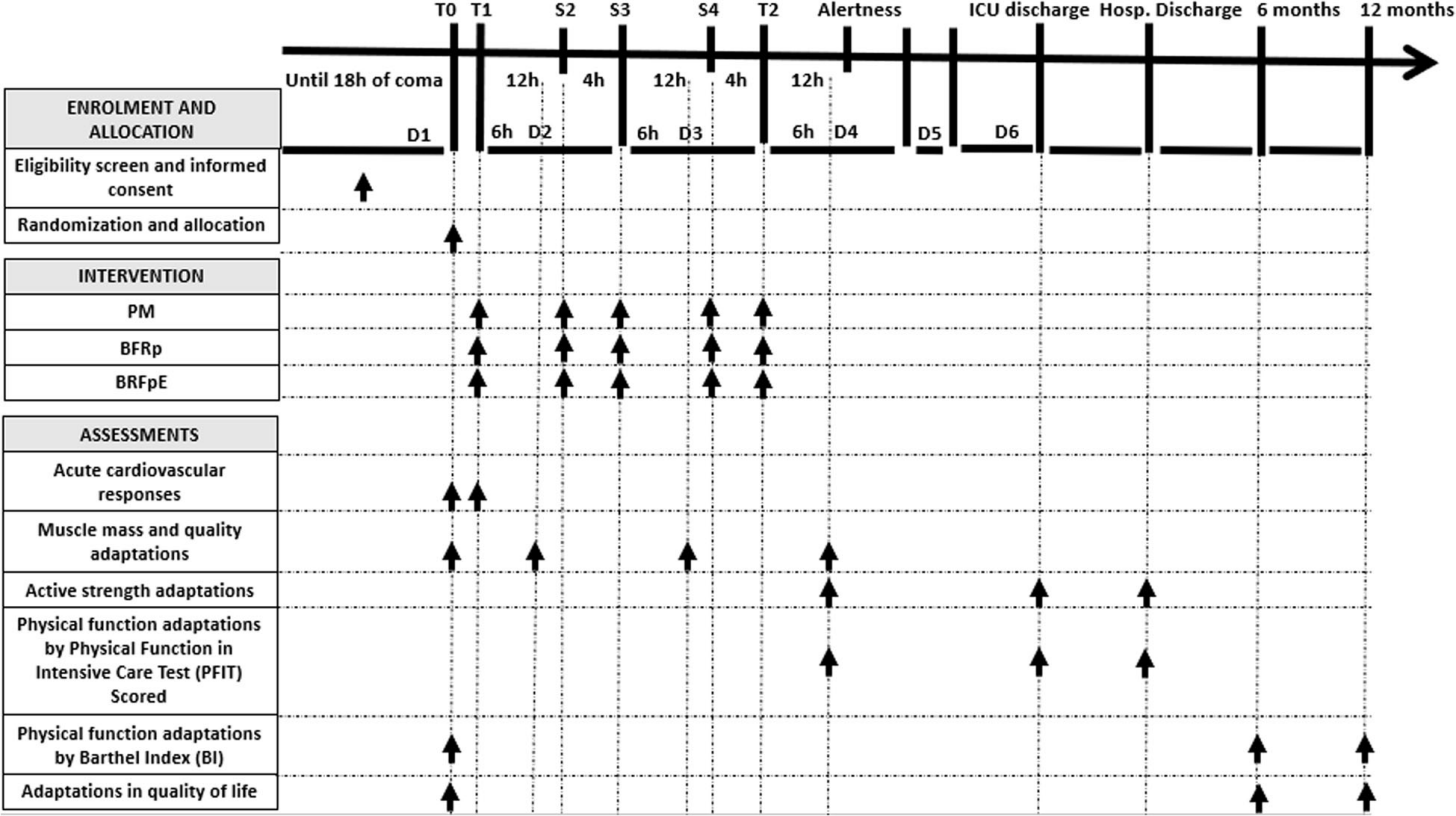
Schedule of enrollment, interventions, and assessments of a patient with 6 days of ICU permanence. ICU, intensive care unit; Hosp., hospital; T0, baseline time point—initial evaluation; T1, time point 1—evaluation during the first training session; S2, session 2—second training session; S3, session 3—third training session; S4, session 4—fourth training session; T2, time point 2—last training session; D1, day 1; D2, day 2; D3, day 3; D4, day 4; D5, day 5; D6, day 6; PM, passive mobilization; BFRp, blood flow restriction associated with passive mobilization; BRFpE, blood flow restriction associated with passive mobilization and combined with neuromuscular electrical stimulation

**Fig. 2:**
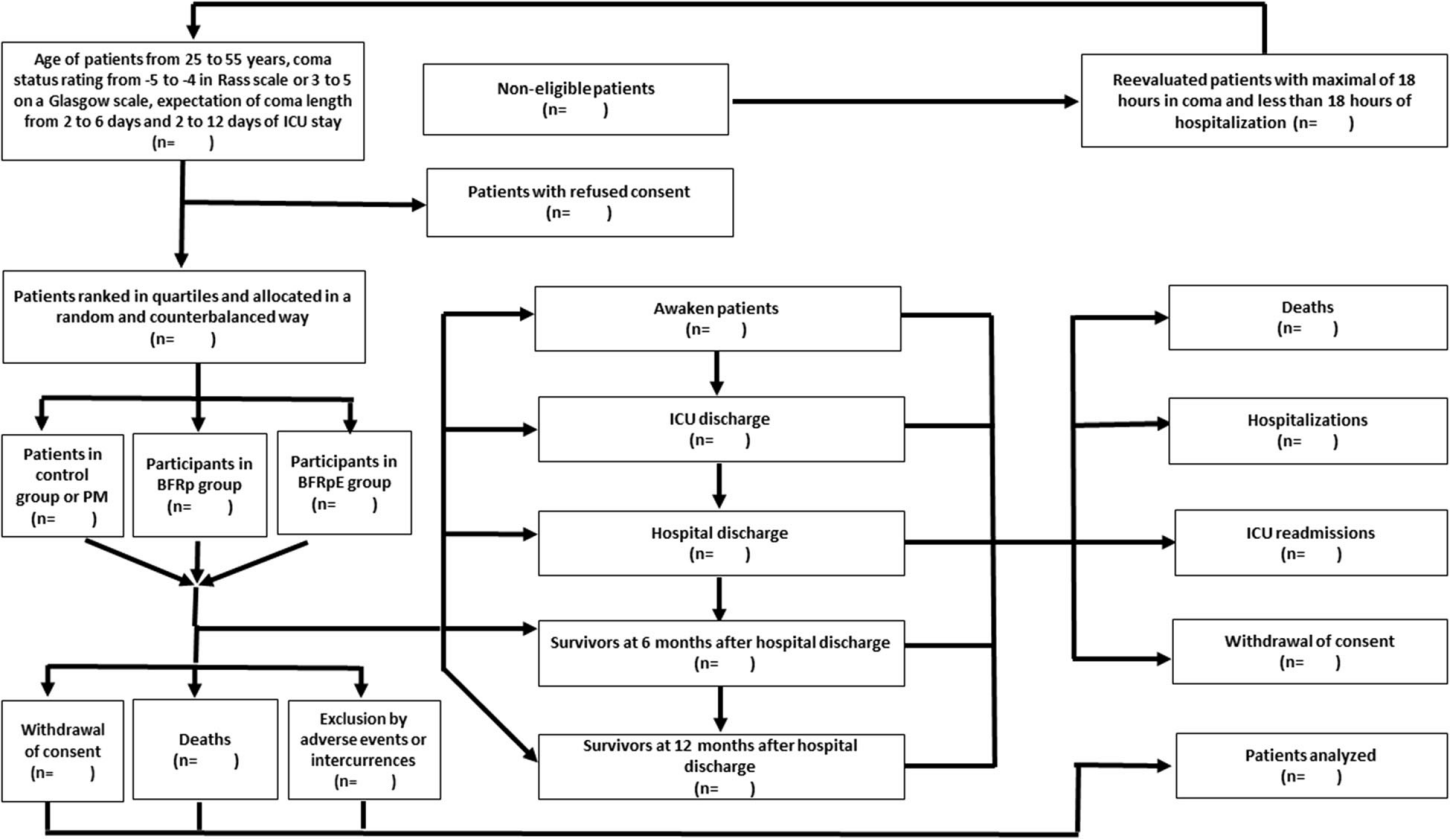
Flowchart of the pilot clinical trial. ICU, intensive care unit; PM, passive mobilization; BFRp, passive mobilization associated with blood flow restriction; BFRpE, passive mobilization associated with blood flow restriction and combined to neuromuscular electrical stimulation

This study describes a protocol for a pilot study of a double-blind, randomized controlled clinical trial of applicability and intervention, with transverse temporality and a three-arm factorial intervention design in accordance with the Standard Protocol Items: Recommendations for Interventional Trials (SPIRIT) guidelines*.* The SPIRIT verification list can be found in the Additional File 1 and is represented in Fig. 1. The flowchart for the clinical trial is shown in Fig. 2.

### Study setting

The clinical trial will be carried out in the teaching and research ICU at the *Irmandade da Santa Casa de Misericórdia de São Carlos*, located in the city of São Carlos, São Paulo, Brazil. This ICU consists of 10 beds, and there is an average of 40 patients admitted monthly. Adult clinical and surgical patients with all types of diseases are treated in the ICU, and the average length of stay is 8 days.

### Sample size rationale

A total of 30 participants were used for the sample size calculation of the clinical trial [43]. Therefore, a sample of 39 patients will enable 10% sample loss for each experimental group (PM/control, BFRp, and BFRpE). Based on a recruitment average of 24 patients per month, 20% of non-consent and 30% of non-previewed death or immediate discharge, we aimed to recruit 7.2 patients per month. The recruitment in a single ICU will allow the study to be completed in a minimal period of 6 months.

### Study population

The inclusion, non-inclusion, and exclusion criteria are shown in Table 1. All the criteria will be reevaluated before each training session, according to the clinical specificity of the criteria (Table 1). All non-included patients will be re-evaluated after 18 h of coma, except for patients whose hospitalization is longer than 18 h prior to ICU admission. In conclusion, all patients will be assisted by the ICU physiotherapy team. Additionally, the number of PM sessions or any type of physical therapy intervention in the peripheral muscle will be quantified to control the total volume of stimulus for each patient.

**Table 1:** Inclusion, non-inclusion, and exclusion criteria of the study population

Inclusion criteria	Non-inclusion criteria	Exclusion criteria
1. Age of 25 to 55 years	1. Persistent arrhythmia with atrial fibrillation	1. Obese (BMI > 30 kg/m^2^)
2. Scores − 5 or − 4 according to the Richmond Agitation-Sedation Scale (RASS) or 3 to 5 in the Glasgow Comatose Scale	2. Pre-existing atrioventricular block or ventricular tachycardia	2. More than one thromboembolism risk factor
3. Maximal 18 h of coma in ICU admission or during critical care hospitalization	3. Recent acute myocardium infarction	3. Previous venous thrombosis and/or pulmonary embolism
4. Prediction of coma length between 2 and 6 days	4. Provisional or definitive pacemaker	4. Peripheral arterial vascular disease history or suspicion of lower limb arterial insufficiency
5. Prediction of 2 to 12 days of ICU permanence	5. Signals of intracranial hypertension	5. Severe cardiovascular instability and/or use of vasoactive drugs in infusion ≥ 0.5 mcg/kg/min
6. Daily protein consumption standardized for the ICU medical and nutrition teams	6. Neoplasia in chemotherapy treatment	6. Deep coagulopathy (prothrombin time (PT) > 2.5 times the normative values, activated partial thromboplastin time (APTT) > 2 times the normal or platelet count ≤ 50.000/μl)
	7. Non-consolidated fractures	7. Heparin intravenous infusion ≥ 2 UI/ml
		8. Fever or hypothermia
		9. Anemia
		10. Neurodegenerative disease
		11. Musculoskeletal disease

*ICU* intensive care unit

### Experimental design

#### Recruitment procedures

Initially, all hospitalized patients in the ICU will be assessed according to the inclusion criteria of the study. After screening, the legal representative will be interviewed and invited to be a participant in the study. Following consent, the legal representative will answer the functional performance of the basic activities of daily living through the Barthel Index [44] and the quality of life through the Medical Outcome Study 36 – Item Short Form Health Survey (SF-36) questionnaire [45, 46]. Following that, the patient will undergo other initial evaluations (T0) within a 23-h period after admission to the ICU or coma identification in the ICU hospitalization for the baseline demographic characterization, clinical, cardiovascular, and neuromuscular.

#### Randomization procedures

Initially, all participants will be pooled into a single group. Afterwards, patients will be classified into quartiles by muscle thickness (MT) of *vastus lateralis* (VL) and type of critical disease and assigned in a random and counterbalanced way into one of the three training protocols: PM/control, BFRp, or BFRpE using a maximally tolerated imbalance model [47]. MT will be measured exclusively in the right leg. An unpaired *t* test will be used to ensure that there is no baseline difference between the groups. If significant between-group differences are detected, randomization will be repeated until a random distribution with no difference is achieved [48]. The Berger-Exner test will be applied to verify selection bias [49].

#### Primary outcome measurements

##### Acute cardiovascular responses

The first training session (T1) will immediately succeed T0 and will have the monitoring of acute cardiovascular responses to training. Mean arterial pressure (MAP), central and peripheral systolic blood pressure (SBP [SBPc and SBPp]), and central and peripheral diastolic blood pressure (DBP [DBPc and DBPp]) will be measured by pulse wave analysis. Concomitantly, the heart rate (HR) and peripheral oxygen saturation (SpO_2_) should be recorded through a portable vital signal monitor (Dixtal, DX 2021, Amazonas, Brazil) every 2 min. The mean of 5 recordings at rest (10 min of rest), 18 recordings during exercise (~ 37 min of training), and the average of 5 post-exercise recordings (10 min) will be considered in the analysis. Electrocardiographic signals will be monitored, and, in case of adverse events, the session will be promptly interrupted. The training protocol will be applied to both legs during the session, simultaneously and alternately between the stimulated leg and the rest of the contralateral limb [37].

##### Cardiovascular safety and applicability of peripheral muscle training

To assess cardiovascular safety, HR values < 50 bpm or > 180 bpm, SBP < 80 mmHg or > 160 mmHg, DBP < 60 mmHg or > 80 mmHg, MAP < 60 mmHg or > 120 mmHg, cardiac or respiratory arrythmias, SpO_2_ < 88%, signals of respiratory discomfort [50, 51], or signals of deep venous thrombosis will be considered criteria to interrupt the protocol. Except for the occurrence of deep venous thrombosis, two consecutive training attempts will be performed after the first training interruption before withdrawing the patient from the study. It is suggested for two main reasons: to exclude the possibility of an occasional adverse event due to the variability of the clinical conditions, and to maintain the training frequency in case the patient continues in the study. In case of exclusion, the protocol will be considered unsafe for this patient. However, in case 15% or more unsafe conditions occur in the same training protocol, the protocol will be excluded from the study. To evaluate the applicability, the number of adverse events and intercurrence will be recorded, and if a total number of adverse events and/or intercurrence overcome 20% of the total training sessions, or there is deep venous thrombosis occurrence in at least 5% of the patients, protocols will be considered unsafe and impracticable. If cardiovascular variations are greater than 20% of the baseline, the protocol will be considered impractical.

#### Measurement of secondary outcomes

##### Neuromuscular adaptations of muscle mass and strength

Muscle mass and quality adaptations will be assessed daily prior to the first training session (~ 12 h from the last session) and 12 h after the last training session (T2) (until 12 h prior to the coma awakening), to the predicted death or ICU discharge. The muscle mass of the VL, *rectus femoris* (RF), and anterior tibial (AT) will be measured by MT and muscle quality, by echo intensity, both analyses obtained by ultrasound images. If the patient awakes from a coma and has the appropriate cognition to collaborate in active assessments, the global muscle strength will be evaluated by the Medical Research Council (MRC) score after 12 h of T2, in the ICU and at hospital discharge. Adequate cognition will be assessed by the absence of delirium after completing the Confusion Assessment Method for Intensive Care Units (CAM-ICU) score [52].

##### Physical function adaptations

Physical function will be assessed 12 h after T2 and in the ICU and at hospital discharge through the Physical Function in Intensive Care Test Scored (PFIT). Lastly, during home follow-up, functional motor performance of basic activities of daily living will be evaluated using the Barthel Index, 6 and 12 months after hospital discharge, by telephone.

##### Quality of life adaptations

Quality of life will be measured simultaneously with basic activities of daily living performance, using the SF-36 questionnaire, which is validated in Portuguese and Brazilian culture and is reliably applicable by telephone [53, 54].

#### Blinding procedures

To be considered a double-blind study, one of the authors will be assigned to create codes for the training-related adaptation data, in addition to being responsible for the randomization of patients. The evaluator, who evaluates the safety and training-related adaptations, will be blinded to the experimental groups. Each experimental group will be led by a different trainer who will also be blinded for the other training groups. Lastly, participants will be blinded by their coma, and in case the participant awakes from coma, information from the study will be provided, but not about the protocol they had previously undergone.

### Study interventions

#### Peripheral muscle training

After T1, in view of the cardiovascular safety and applicability of PM, BFRp, and BFRpE, training sessions will be held twice a day, divided by 4 h [51]. The starting training leg will be randomly chosen at T1 and, from that, alternated in every training session. Patients will undergo the experimental protocol if they present controlled temperature (between 36.5 and 37.5 °C), stable blood pressure (> 100 and < 150 of SBP and > 60 and < 100 of DBP), SpO_2_ > 90%, respiratory rate (RR) < 25 rpm, and HR > 60 and < 140 bpm [55].

##### Passive mobilization/control group protocol (PM)

The PM protocol will be modified from Barbalho et al. [37]. The authors applied 3 sets of 15 mobilizations for flexion and extension of the hips, knees, and ankles. Movement was standardized in 2:2 using a metronome (2 s flexion and 2 extension), totaling 3 min of PM. To standardize PM with the BFR protocol, in every minute of PM, 1 min of rest will be allowed, totaling 5 min of PM for each set. Following that, a 3-min rest will be allowed, and the mobilization will be repeated in 5 sets. The session will last 37 min on average [56].

##### Passive mobilization associated with blood flow restriction protocol (BFRp)

A pressure cuff with 17.5 cm of width and 94 cm of length (JPJ, Sao Paulo, Brazil) with a pressure manometer will be placed on the quadriceps muscle, on the inguinal fold. Afterward, a vascular Doppler probe (DV-600; Martec, Ribeirao Preto, Brazil) will be placed in the posterior tibial artery. To determine the occlusion pressure, the cuff will be inflated until the auscultatory pulse stops [57]. An external compression of 85% of the total vascular occlusion pressure will be applied for 5 min, followed by a 3-min rest (compression release) [42]. The BFR protocol will be synchronized to the PM protocol. Compression will be repeated 5 times during the training session, and occlusion pressure will be re-evaluated in all sessions.

##### Passive mobilization associated with blood flow restriction and combined with neuromuscular electrical stimulation (BFRpE)

The procedures for BFRp will be adopted and synchronized with an electrical stimulation protocol. For the electrical stimulation electrode placement, the skin will be subjected to trichotomy, abrasion, and asepsis. Rectangular-shaped electrodes (50 × 50 mm) will be placed on the distal and proximal motor points of the VL (proximal and distal points located between the reference line of the antero-superior iliac spine and the superior lateral border of the patella and on the line between the apex of the great trochanter and the superior lateral border of the patella, respectively), RF (located on the reference line between the antero-superior iliac spine and the superior patella border), and AT (on the line between the apex of fibular head and the medial malleolus). In addition to the reference lines, the placement of the electrodes demonstrated in Santos et al. [58] and Dirks et al. [59] will be used to guide the location and electrostimulation of VL, RF, and TA. Additionally, this electrode placement will be marked with a semi-permanent pen to maintain the stimulus points throughout the study. A biphasic electro stimulator (Dualpex 071, Quark Medical, Piracicaba, Brazil) will provide symmetric pulses of 100 Hz, pulses lasting 400 μs, 5 s on (0.75 s rise, 3.5 s contraction and 0.75 s decay), and 10 s off, the intensity must be capable of causing visible contractions. The mean intensities used should be maintained from 29 to 33 mA and increased in approximately 3 min, when muscle contraction is no longer visible. The session will last 40 min, including 30 min of stimulation, a 5-min warm-up, and a 5-min cool-down, both with 5 Hz of frequency, pulse wave lasting 250 μs, and sub-maximal intensities (without muscle contraction) [59, 60]. The same researcher will apply both the BFRp and electrical stimulation.

### Cardiovascular assessment

#### Cardiovascular assessment by the pulse wave analysis

Pulse wave analysis will be performed using SphygmoCor equipment (SphygmoCor, AtCor Medical, Sydney, Australia). In a supine position, the patient will have a pneumatic clamp placed in the proximal portion of the left arm. Following that, additional data such as age, weight, and height will be provided to the system. After a 10-min rest, three measurements of systolic and diastolic arterial pressure will be performed in a 2-min interval [61] and accepted as long as the difference is less than 10% [62], or 5 mmHg [63] between the measurements. Afterward, the mean value of the three accepted measurements will be inserted into the system to obtain the MAP, SBPc, SBPp, DBPc, and DBPp [64–67]. In T0, the variables will be measured after 10 min of rest. In T1, it will be taken prior (10 min), during (23 and 37 min), and after (47 min) the training session. The average between minutes 23 and 37 (pause between sets) will be used as the exercise value.

### Muscle mass and quality assessment

#### MT and echo intensity (MT_echo_) by ultrasonography

MT and MT_echo_ will be measured by ultrasound images (Mysono U6 EX; Samsung Medison, Gangwon-do, South Korea). For body fluid homogenization, 15 min of rest in the supine position will be allowed, with the lower limbs neutrally positioned and sustained by a Velcro strip. A 7.5 MHz linear-array probe (LN5-12; Samsung Medison, Gangwon-do, South Korea), revested by surface gel, to promote acoustic coupling without dermic compression, will be placed longitudinally to the muscle fibers. The anatomic reference between two MT points of each muscle will be defined by the distal motor point sites used for the electrical stimulation. Additionally, for the VL, the anatomical reference will be the middle portion between the great trochanter and the lateral epicondyle [68]. For RF, the reference will be set at 50% and at two-thirds of the distance from the anterior superior iliac spine and the superior patella border [51, 69]. Anatomical references for AT will be defined in one-fourth of the distance between the inferior portion of the patella and the lateral malleolus and one-third of the distance between the border of the fibula and the medial malleolus [70]. Sequential images will be acquired with 6 cm of depth and, after digitalization, analyzed using the ImageJ software (National Institute of Health, USA). MT (cm) will be characterized as the perpendicular measurement between the superficial and deep aponeurosis [71]. MT_echo_ (A.U.) will be evaluated using greyscale grading, obtained by the histogram function of the software (0 = black and 256 = white) [72]. Three consecutive images of the same anatomic point will be analyzed, and the mean will be adopted as the final value for MT and MT_echo_ [73]. MT_echo_ will be normalized by MT (i.e., MT_echo_/MT, cm) to explain the effects of alterations on muscle size [57].

### Muscle strength assessment

#### Global muscle strength by MRC score

Six muscle groups of the upper and lower limbs will be assessed bilaterally through the global strength (shoulder abductors, elbow flexors, wrist extensors, hip flexors, knee extensors, and feet dorsiflexors) by the MRC score [51, 74, 75]. Test familiarization will be performed through passive movements, and the tests will be applied in a fixed order. All muscle groups will be rated from 0 to 5, where 0 means absence of visible/palpable muscle contraction and 5 means movements performed against gravity and resistance [75]. For the fragile and critical population, patients will be positioned in dorsal decubitus with the headboard angled at 45°. Then, arm abduction will be tested against gravity with the elbow in flexion. In case of a successful attempt, the strength will be rated from 4 to 5. Otherwise, patients will be repositioned in dorsal decubitus at 10°. to be rated from 0 to 3. Following that, the elbows, wrists, hips, knees, and ankles will be tested. In case any muscle group cannot be evaluated due to a central/peripheral nervous injury or orthopedic problems, the total sum will be calculated by extrapolating the identical contralateral muscle [20]. The weakness acquired from the ICU will be identified from scores < 48 [76] and classified as severe weakness if < 36 points [75].

### Physical function assessment

#### Physical function by the PFIT scored

The PFIT scored will be applied to measure the physical function of the ICU patients. The score presents four items (assistance with sit to stand, cadence, strength of the shoulder flexors, and strength of the knee flexors), and for each item, the scoring varies from 0 to 3. The maximal score is 12, which indicates functional independence [77]. The PFIT scored will be applied through the translated and validated version for the Brazilian population [78].

#### Physical function by the Barthel Index

The Barthel Index is widely used in Brazil [79, 80] and is applied in severely ill patients [81–83] and administered by telephone [84, 85]. It comprises 10 items weighted differently. Two items (bathing and personal care) can be graded in two alternatives (0 and 5 points), whereas 6 items (feeding, dressing, bowels [fecal and urinary continence], toilet use, stairs), in three alternatives (0, 5, and 10 points), and two items (transfers and mobility) present four alternatives (0, 5, 10, and 15 points). The final score is obtained by summing each item, varying from 0 (complete dependence) to 100 (independence) [44].

### Quality of life assessment

#### Quality of life using the SF-36 questionnaire

Quality of life will be assessed using the SF-36 questionnaire, an instrument of good acceptance, reliability, and validity for ICU patients validated in Brazil and easily administered by telephone [45, 46, 53, 54]. It comprises 8 domains: physical function, role-physical, bodily pain, general health, vitality, social functioning, role-emotional, and mental health. Scores range from 0 to 100, where 0 corresponds to the worst general health status, and 100 corresponds to the best health condition [86–88]. The categorization and description of the methods and analyses are shown in Table 2.

**Table 2:** Categorization and description of the methods and analyses of the study variables

Evaluation category	Organic responses	Method of evaluation	Variables in analyses
Safety and applicability	Acute cardiovascular	Pulse wave analysis (PWA)	MAP, SBPc, SBPp, DBPc, and DBPp (mmHg)
Safety and applicability	Acute cardiovascular	Analyses of the vital signs	HR (bpm) and SpO_2_ (%)
Muscle systems	Muscle mass adaptation	Ultrasound image	Muscle thickness (cm)
Muscle systems	Muscle quality adaptation	Ultrasound image	Echo intensity (A. U. from 0 to 256)
Neuromuscular systems	Global muscle strength adaptations	Medical Research Council (MRC) Score	Number scoring (0 to 60)
Physical function	Adaptations in physical function in ICU	Physical Function in the Intensive Care Test Scored (PFIT)	Number scoring (0 to 12)
Physical function	Adaptations in physical functional performance after hospital discharge	Performance in the basic activities of daily living by the Barthel Index (BI) by telephone interview	Number scoring (0 to 100)
Clinical outcomes	Adaptations in quality of life	Medical Outcome Study 36 – Item Short Form Health Survey (SF-36) questionnaire	Number scoring (0 to 100)

*SBPc* central systolic blood pressure, *SBPp* peripheral systolic blood pressure, *DBPc* central diastolic blood pressure, *DBPp* peripheral diastolic blood pressure, HR heart rate, *MAP* mean arterial pressure, *SpO*_*2*_ peripheral oxygen saturation, *ICU* intensive care unit

## Statistical analysis

After data collection, participants will be distributed into subgroups by periods in coma (2, 3–4, 5–6 days), in alertness (1–2, 3–4, 5–6 days), and in ICU permanence (3–4, 5–6, 7–8, 9–10, 11–12 days). The normality of data will be analyzed using the Shapiro-Wilk test. One-way analysis of variance (ANOVA) will be used for intra-group comparisons in T0. A mixed-model analysis will be used for each dependent variable, whereas group and time will be considered as fixed factors and subject as the random factor. In case of significant *F*, the Tukey adjustment will be applied for multiple comparisons. In the event of a non-normal data distribution, a Kruskal-Wallis test will be applied to test for the differences in baseline values and the Friedman test to analyze the repeated measures. The adjusted *p* values will be calculated using the Hommel procedure [48]. Effect sizes between and within groups will be calculated using Cohen’s *d* [89]. The minimal clinically important difference will be assessed with distribution-based methods [90]. The statistical significance will be assessed at *p* < 0.05. All analyses will be performed using R 3.6.2 (The R Project for Statistical Computing, 2019) in R-studio 1.3.443 (RStudio Inc., Boston, MA, USA).

## Discussion

The present study focuses on describing an innovative experimental protocol based on cardiovascular safety, scope of research, and distinct experimental control to compose a randomized controlled clinical trial, which aims to investigate the applicability and the effect of a novel combination of training protocols of peripheral muscle atrophy and weakness, functional adaptations and on quality of life in severely ill comatose patients and within after 12 months of hospital discharge.

The innovative initial aspects of the study involved the purpose of optimizing the combination of training protocols for severely ill comatose patients. The first protocol consisted of an adaptation of a recently applied protocol, comprising PM and BFR [37] adjusted to the current BFR recommendations [42], aiming to reduce muscle atrophy and weakness. The second purpose included a novel combination involving electrical stimulation, which is widely applied in ICUs [7, 8], with PM and BFR [37] to prevent muscle atrophy and weakness and maximize the functional adaptations and the quality of life, as well as its return to household and social activities.

In addition to using an innovative combination, the experimental design was modified from the intra-subject design, used by previous studies in the field such as Dirks et al. [59], Barbalho et al. [37], and Chhetri et al. [51], to an inter-subject design. The inter-subject design enables the application of protocols bilaterally, simultaneously, and alternately, which allows the replication of the intensive care clinical practice and preserves the ecological validity and investigation of the maximized effects of training on muscle atrophy and weakness, functional responses, and outcome of quality of life of ICU coma surviving patients.

By replicating the intensive care practice, a combination of PM, BFR, and electrical stimulation applied bilaterally, simultaneously, and even alternately will promote unknown cardiovascular responses, which may be investigated as a guarantee of vital safety of the critical patients during the physiological stress imposed by the exercise. Based on that, the present study aimed to measure the cardiovascular responses during and after the first training session, despite the absence of intercurrence or adverse events reported during passive BFR and BFRp, or electrical isolated stimulation [37, 59].

Concerning the scope of the investigation, in addition to the daily assessment of muscle mass, we also proposed a broad assessment of the strength, physical function, and quality of life of the ICU coma surviving patients. Thus, we suggest not only assessing the isolated muscular system in the ICU, but also monitoring the functional integration of the systems through functional analysis and the outcome of quality of life.

For muscle mass, the option of measuring the MT by ultrasound images was based on the scientific validation of this method, which is quicker and simpler for bedside conditions than the cross-sectional area assessment [91]. Additionally, MT presents lower measurement errors such as the coefficient of variation and the typical error, when compared to the muscle cross-section area. The elevated coefficient of variation and typical errors demonstrate high variability between two consecutive measurements, which precludes the detection of daily changes in muscle mass in comatose patients. As a consequence, non-detection of slight changes could impair the management of muscle atrophy prevention, and therefore, the clinical evolution of the patients [92].

In addition to the assessment of muscle mass, muscle quality was evaluated using the MT_echo_ analysis. This method consists of classifying the ultrasound images through greyscale grading, which varies from 0 to 256, where 0 means low gray coloration and 256, intensive gray coloration and characterizes the presence of edema, fat or healthy muscle tissue [72]. Thus, in addition to muscle mass, ultrasound images can assess muscle quality, which involves not only muscle fibers, but also the presence of inflammatory liquid and/or intramuscular fibrosis acquired from low mobility changes, intensive therapy strategies, and exercise-related muscle damage. Additionally, muscle quality assessment allows normalization of MT to the respective echo intensity. This strategy overlooks changes in edema or fibrosis in the MT results, suggesting that the promoted changes are mainly due to the real effect of resistance training on muscle mass [93].

To test muscle strength, the MRC score was applied, which is the gold standard assessment for global strength [74]. This method was suggested to evaluate the muscle strength of patients that awake from coma until hospital discharge. Thus, we were concerned with monitoring the neuromuscular evolution of the critical patient, targeting it to analyze the effect of training during the whole hospitalization stay.

Consecutively, the physical function measurement was described to evaluate the interaction of the neuromuscular system with the other organic systems. Thus, we suggested applying the PFIT scored while awake from coma and ICU and hospital discharge. In addition, the Barthel Index was suggested to assess physical performance during the recovery process at home, as it is validated for use through telephone contact [80, 94].

Thus, an evaluation of the clinical outcome after 6 and 12 months of hospital discharge was also structured in the system’s functional integration to evaluate the household and social reinsertion of patients surviving ICU coma. The rationale of our choice was based on associating muscle strength loss with the worsening of quality of life [27, 29–31, 95]. From this, the follow-up of the clinical outcome was suggested through the SF-36 questionnaire.

In addition to suggesting a broad and integral approach to neuromuscular and systemic functional assessments, the inclusion criteria and the experimental design were based on the largest possible number of methodological control conditions. Thus, for the inclusion criteria, the preference for comatose adults was essential for better control of the population in question.

More specifically, our objective was to ensure that the muscular evaluation was performed during the longest period of bed restriction, in which muscle atrophy and weakness occurred [15, 17, 32]. Finally, patient admission was based on a moderate to severe coma classification, aiming to avoid different volumes of active contractions and, consequently, different stimuli in muscle protein syntheses, which would promote a high variability in responses to the study of muscular variables.

Regarding the trial design, the inclusion of patients within the first 18 h of coma identification was suggested to promote a higher experimental control. The aim of this was to ensure that there would be enough time to perform the admission procedures and the first training session, before the beginning of the deleterious effects associated with low mobility [15, 16].

Another control that was adopted was the randomization of patients into experimental groups by ranking the characteristics that could impair the between-group comparison. More specifically, a peculiar aspect of intensive care studies is the high variability of the sample characteristics, principally in adult ICUs, where the admission involves non-pediatric patients of both sexes, with all types of clinical severe illness, pre- and post-surgery. Thus, to homogenize the experimental groups, we purposely stratified the participants into quartiles, considering MT and type of disease.

To further enhance trial control, our study also proposed daily assessments involving muscle mass. This strategy enables a detailed and trustworthy monitoring of the training-related responses, which differs from specific evaluations, performed during the pre- and post-training period. In addition, this strategy may be important for the experimental and statistical adequacy of the clinical trial to intensive care, in which daily pre- and post-session assessments allow lower experimental losses. Thus, in the event of unanticipated patient discharge or death, the statistical analysis may include the last assessment data from the previous day.

Despite the positive aspects presented, the study is not without limitations. The inclusion of patients with varied duration of coma (2–6 days of intervention and 2 to 12 days of ICU permanence) may lead to high heterogeneity at both time points. As an alternative, the use of subgroups for the duration of the coma (2–3, 4–5, and 6 days), alertness (1–2, 3–4, and 5–6), and the time of stay in the ICU (3–4, 5–6, 7–8, 9–10, and 11–12) was suggested. Finally, the patients who were not classified in the study groups or in the added subgroups should be included in the clinical trial flowchart, but not in the statistical analysis.

From the promising and novel optimizing association of early mobilization protocols with comatose patients in the ICU, using broad methods of clinical monitoring and methodological control, the present study promoted a wide, integral, innovative, and viable experimental protocol to a future randomized controlled clinical trial, aiming to reproduce new scientific evidence regarding early mobilization in critical care. More specifically, our study enabled the investigation of cardiovascular safety, applicability, and effects of innovative peripheral motor interventions to prevent muscle atrophy and weakness, reduced physical function, and low quality of life, which were monitored since coma until health recovery with 12 months of hospital discharge.

Thus, we expect to contribute to the clinical practice of early passive mobilization in critically comatose patients through the purpose of maximizing the effects of an optimized interventional protocol and, consequently, cooperating for musculoskeletal rehabilitation and functional reinsertion in the home and social activities of survivors of coma in ICUs.

## Trial status

This manuscript is based on the most recent version of the protocol (4 April 2020). The study was opened to recruitment at the ReBec site in April 2020, and the first participant was recruited in the same month. Recruitment was expected to be completed in October 2020; however, an insufficient number of participants was recruited due to the current pandemic scenario. In this regard, recruitment was suspended during the pandemic quarantine period in Brazil which explains the delay in submitting the study to this journal.

## Supplementary Information

**Additional file 1.** SPIRIT 2013 Checklist: Recommended items to address in a clinical trial protocol and related documents*.

## Data Availability

The datasets supporting the conclusions of this article are available in the repository of the ReBec (http://www.ensaiosclinicos.gov.br), registration number RBR-2qpyxf.

## Abbreviations

ICU: Intensive care unit
PM: Passive mobilization
BFR: Blood flow restriction
BFRp: Blood flow restriction associated with passive mobilization
BFRpE: Blood flow restriction associated with passive mobilization and combined with electrical stimulation
T0: Baseline
T1: First training session
T2: Last training session
SPIRIT: Standard Protocol Items: Recommendations for Interventional Trials
NRS-2002: Nutritional Risk Screening 2002
SF-36: Medical Outcome Study 36 – Item Short Form Health Survey
MT: Muscle thickness
VL: *Vastus lateralis*
SBP: Systolic blood pressure
SBPc: Central systolic blood pressure
SBPp: Peripheral systolic blood pressure
DBP: Diastolic blood pressure
DBPc: Central diastolic blood pressure
DBPp: Peripheral diastolic blood pressure
HR: Heart rate
MAP: Mean arterial pressure
SpO_2_: Peripheral oxygen saturation
RF: *Rectus femoris*
AT: Anterior tibial
MRC: Medical Research Council
CAM-ICU: Confusion Assessment Method for Intensive Care Units
PFIT: Physical Function in Intensive Care Test Scored
RR: Respiratory rate
MT_echo_: Echo intensity
ANOVA: Analysis of variance
SD: Standard deviation
ReBec: Brazilian Clinical Trial Registry
UFSCar: Federal University of São Carlos
CAPES: Coordination for the Improvement of Higher Education Personnel
LACAP: Cardiopulmonary Physiotherapy Laboratory
MUSCULAB: Laboratory of Neuromuscular Adaptations to Resistance Training
